# Babao Dan attenuates hepatic fibrosis by inhibiting hepatic stellate cells activation and proliferation via TLR4 signaling pathway

**DOI:** 10.18632/oncotarget.12783

**Published:** 2016-10-20

**Authors:** Lei Liang, Xue Yang, Yang Yu, Xiaoyong Li, Yechen Wu, Rongyu Shi, Jinghua Jiang, Lu Gao, Fei Ye, Qiudong Zhao, Rong Li, Lixin Wei, Zhipeng Han

**Affiliations:** ^1^ Tumor Immunology and Gene Therapy Center, Eastern Hepatobiliary Surgery Hospital, the Second Military Medical University, Shanghai, China; ^2^ Medical College of Soochow University, Suzhou, China; ^3^ Department of General Surgery, Yangpu Hospital, Tongji University School of Medicine, Shanghai, 200065, China

**Keywords:** hepatic fibrosis, hepatic stellate cells, chinese medicine, Babao Dan, toll-like receptor 4

## Abstract

Babao Dan (BBD), a traditional Chinese medicine, has been widely used as a complementary and alternative medicine to treat chronic liver diseases. In this study, we aimed to observe the protective effect of BBD on rat hepatic fibrosis induced by diethylnitrosamine (DEN) and explore it possible mechanism. BBD was administrated while DEN was given. After eight weeks, values of serum alanine aminotransferase (ALT) and aspartate aminotransferase (AST) indicated that BBD significantly protected liver from damaging by DEN and had no obvious side effect on normal rat livers. Meanwhile, BBD attenuated hepatic inflammation and fibrosis in DEN-induced rat livers through histopathological examination and hepatic hydroxyproline content. Furthermore, we found that BBD inhibited hepatic stellate cells activation and proliferation without altering the concentration of lipopolysaccharide (LPS) in portal vein. *In vitro* study, serum from BBD treated rats (BBD-serum) could also significantly suppress LPS-induced HSCs activation through TLR4/NF-κB pathway. In addition, BBD-serum also inhibited the proliferation of HSCs by regulating TLR4/ERK pathway. Our study demonstrated that BBD may provide a new therapy strategy of hepatic injury and hepatic fibrosis.

## INTRODUCTION

Hepatic fibrosis, characterized by excessive accumulation of extracellular matrix (ECM) proteins, occurs in most types of chronic liver diseases [1–3]. Advanced liver fibrosis results in irreversible cirrhosis, often accompany with portal hypertension, liver function failure, high susceptibility to infection or to developed hepatocellular carcinoma (HCC) [4–6]. Thus, focusing on events that lead to the primarily accumulation of ECM help to develop new therapeutic targets to attenuate hepatic fibrosis. Quiescent hepatic stellate cells (HSCs) are located in Disse space and characterized by storage of retinoid. Activated HSCs are demonstrated as predominant cell produced ECM to participate in hepatic fibrosis [7, 8]. In addition, activated HSCs regulate the recruitment of inflammatory cells via secretion of chemotactic factors, such as MCP-1. Therefore, inhibition of the HSCs activation and proliferation may be an attractive method to anti-fibrotic therapy [9].

Toll-like receptors (TLRs) were originally identified as pathogen-associated molecular pattern recognition receptors (PRR) and recognized exogenous ligands in response to infection [10]. TLR4, in particular, conferred by the lymphocyte antigen 96 also known as MD-2, can specially response to bacterial lipopolysaccharide (LPS) [11]. The process of presentation LPS to MD-2 can also be facilitated by CD14 or the LPS-binding protein (LPB) [12]. LPS, located in the cytoplasm of Gram-negative bacteria, can be considerable absorbed in liver with increased permeability of the intestinal mucosal barrier in cirrhosis rats or patients [13]. Importantly, LPS can enormously induce HSCs activation and aggravate liver fibrosis through TLR4 pathway, which has been proved to be an important mechanism in liver injury [14]. During HSCs activation induced by LPS, TLR4 signal was activated via an adaptor molecule MyD88, leading to translocation of nuclear factor kappa B (NF-κB) with consequent of transition HSCs to myofibroblasts, and up-regulation of pro-fibrogenic and pro-inflammatory cytokines [15, 16]. Meanwhile, LPS can increase the expression of extracellular-related kinase (ERK) phosphorylation, which can regulate cell proliferation by Cyclin D1 or c-myc [17–20]. It has been demonstrated that TLR4-mutation mice displayed a profound reduction in hepatic fibrogenesis [15]. Thus, TLR4 inhibition can obviously down-regulate inflammation and fibrosis [21, 22]. These results confirm the critical role of TLR4 signaling in regulating HSCs activation and proliferation, which affect the progression of hepatic fibrosis.

Babao Dan (BBD), a mixed powder of traditional Chinese medicine containing eight constituents, including natural calculus bovis, snake gall, antelope horn, pearl, musk, radix notoginseng and so on. The formula of BBD was protected by Chinese Food and Drug Administration (CFDA). It has been widely used as a complementary and alternative medicine to treat chronic liver diseases. However, the function and mechanism of BBD in treating hepatic fibrosis are still unclear. In this study, we demonstrated that BBD can ameliorate liver injury and fibrosis in rat hepatic fibrosis model induced by diethylnitrosamine (DEN), and have no obvious side effect in normal rat livers. We also found that BBD did not influence the absorption of LPS in liver by analysing serum from portal vein. Meanwhile, we firstly illustrated BBD can inhibit LPS-induced HSCs activation and proliferation *in vitro* through TLR4/NF-κB and TLR4/ERK signaling pathway, respectively. Upon our results, BBD may be a novel therapeutic choice for hepatic fibrosis.

## RESULTS

### BBD ameliorated liver injury and the expression of inflammatory cytokines

**Figure 1:**
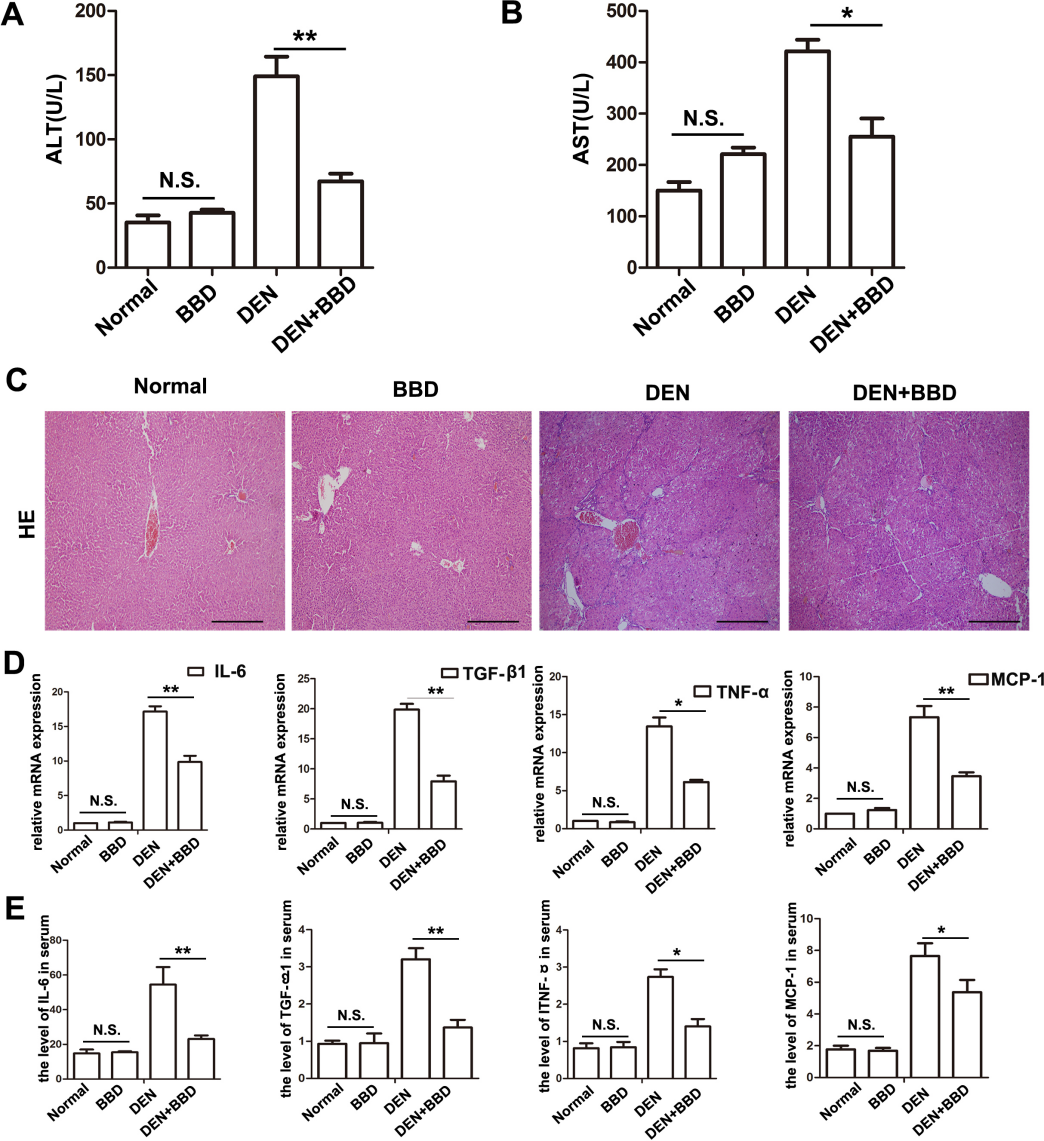
BBD ameliorated liver injury and inflammation in DEN-injured rats DEN was used to build a fibrotic model for studying the effect of BBD. Peripheral vein serum of each rat was collected at 8th week. Then, the levels of ALT (**A**) and AST (**B**) were measured to reflect liver injure. (**C**) HE staining (× 200) was performed to reflect hepatocyte injured and inflammatory cells infiltration. (**D**) The expression of inflammatory cytokines were abstracted from each fresh liver tissue and measured by RT-PCR. (**E**) The level of inflammatory cytokines in peripheral vein serum was also analyzed by ELISA. The unit is ng/L. Compared to DEN group, *P < 0.05; **P < 0.01; compared to Normal group, NS: no significance, by two-tailed Student's *t* test, *n* = 8, bar = 1 mm.

Hepatic fibrosis is the pathological consequence of chronic liver injury. We established a diethylnitrosamine (DEN)-induced hepatic fibrosis model in rats. BBD was gavaged at a dose of 0.5 mg/kg bw, once every two days at the same time of DEN administration. To assess the protective effect of BBD, serum ALT and AST, as an index to evaluate liver injury, were determined after 8 weeks. The results of aminotransferases test presented that DEN enormously elevated the concentrations of ALT and AST, while BBD significantly decreased them. In addition, the result also indicated BBD had no obvious damage to normal rat lives. (Figure 1A, 1B). We next examined the potential effect of BBD on hepatic inflammatory infiltration. Liver paraffin sections stained with H&E presented that DEN severely damaged rat livers at 8th week and BBD obviously reduce hepatocyte damage and inflammatory cells infiltration (Figure 1C). At the same time, the expression of inflammatory cytokines such as interleukin 6 (IL-6), transforming growth factor (TGF)-β1, tumor necrosis factor (TNF)-α and monocyte chemotactic protein (MCP)-1, in fresh liver tissues were detected by RT-PCR (Figure 1D). Meanwhile, the expression of inflammatory cytokines in peripheral vein serum was also detected by ELISA (Figure 1E). Results of RT-PCR and ELISA indicated that contrast to DEN-group, BBD obviously decreased inflammatory cytokines expression. Importantly, the date also indicated BBD had no or non-significant damage on normal rat livers. Consequently, the results had determined that BBD could protect hepatocytes from DEN-injury, and inhibited inflammatory infiltration and inflammatory cytokines expression.

### BBD attenuated hepatic fibrosis

**Figure 2:**
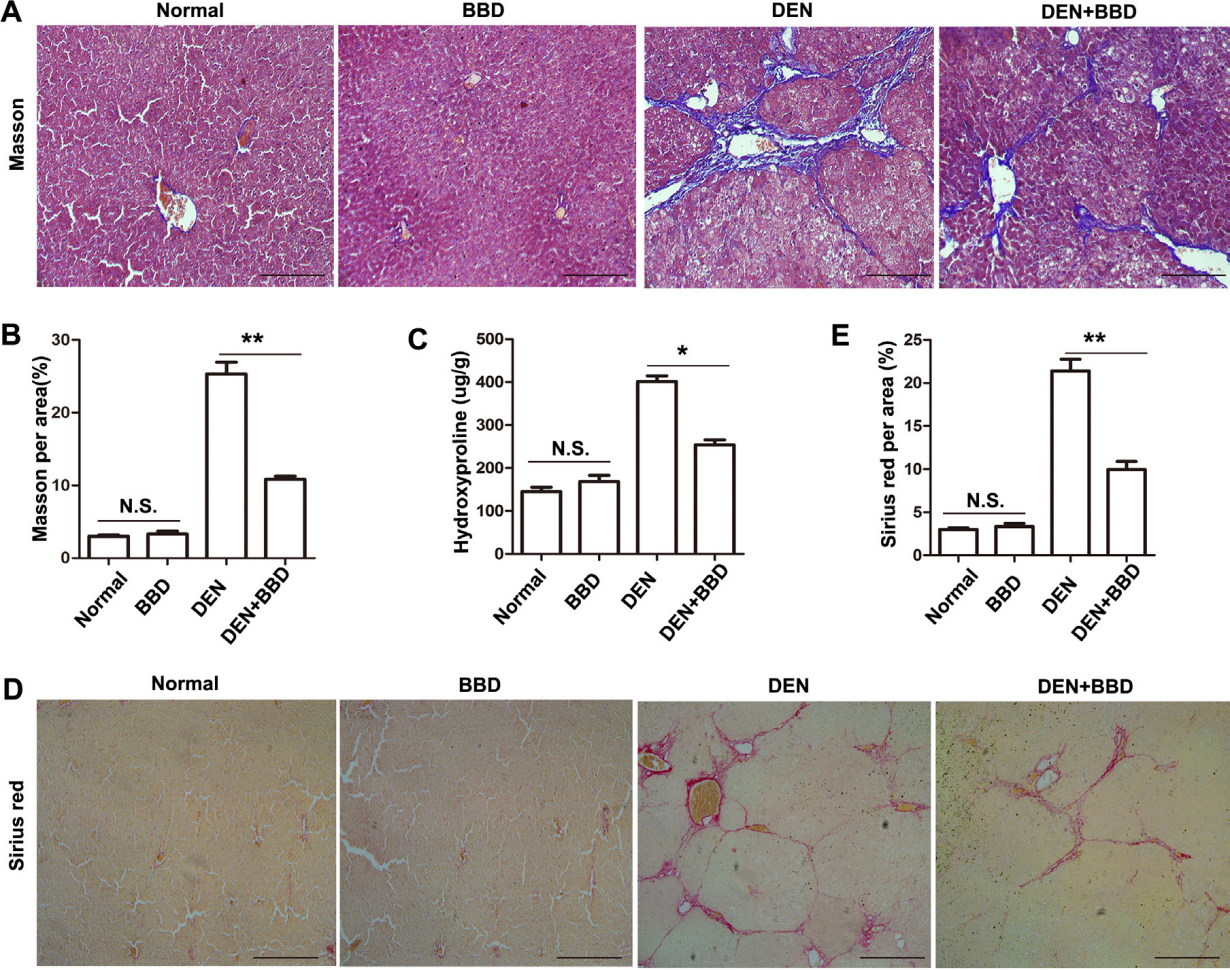
BBD attenuated hepatic fibrosis 4-μm-thick paraffin-embedded liver section was using for histopathological examination. (**A**) The deposition of collagens was stained by Masson'trichrome (× 200) and the stained area was quantified using an image analysis system (**B**). (**C**) The contents of hydroxyproline, one of the main content of collagens, were tested according the protocol in Hydroxyproline Testing Kit. (**D**) Sirius red (× 100) was stained for hepatic fibrosis. (**E**) The percentage of Sirius-red in fibrotic livers was quantified using an image analysis system. Compared to DEN group, *P < 0.05; **P < 0.01, compared to Normal group, NS: no significance, by two-tailed Student's *t* test, *n* = 8, bar = 1 mm.

We next explore the influence of BBD on hepatic fibrosis. Masson's trichrome stain was performed to assess deposition of collagen. Compared with DEN-group, DEN with BBD group obviously attenuated the deposition of collagen in livers, and the area of stained by Masson was significantly reduced 57.2% (Figure 2C, 2D). Meanwhile, the content of hydroxyproline, one of the main content of collagen, in BBD-treated rats with DEN administration was lower than that in the DEN-induced rats (*n* = 8, 254.14 ± 25.58 μg/g vs. 401.34 ± 30.53 μg/g, *P* < 0.05) (Figure 2D). In addition, Sirius red stain also presented that periportal fibrosis with fibrous septa extended to adjacent portal tracts and the terminal hepatic venue in the DEN-group and BBD significantly reduced the deposition of ECM. In contrasted to DEN-induced rat livers, the area of fibrosis was significantly reduced 53.6% after followed with BBD administration (Figure 2E). Furthermore our date show that BBD had no obvious side effect in normal rat livers. As a result, the date suggested that BBD does could ameliorate hepatic fibrosis induced by DEN in rats.

### BBD inhibited hepatic stellate cells activation through inhibiting TLR4/IKK/NF-κB signaling

**Figure 3:**
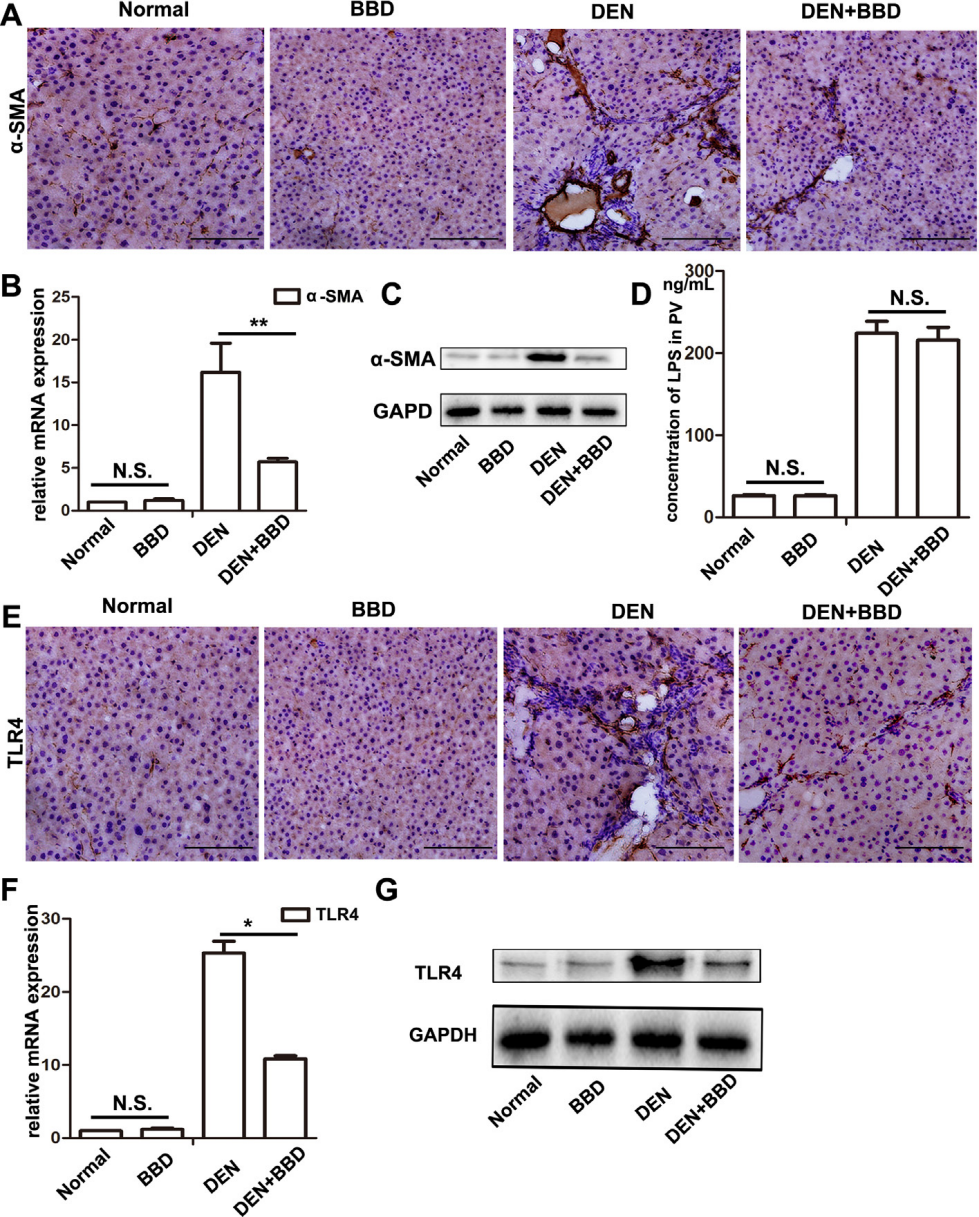
BBD inhibited hepatic stellate cells activation and the expression of TLR4 without attenuating the concentration of LPS 4-μm-thick paraffin-embedded liver section was using for immunohistochemical staining (IHC). (**A**–**C**)The protein expression of α-SMA (× 200), a marker of activated HSCs, was analyzed by IHC, and quantitative measured by RT-PCR and Western blot. (**D**) Concentration of LPS in portal vein serum was detected by Rat endotoxin ElISA test kit. (**E**–**G**) The protein expression of TLR4 (× 200), a receptor specially bind to LPS, was determined by IHC, and also quantitative measured by RT-PCR and Western blot. Compared to DEN group, *P < 0.05; **P < 0.01, compared to Normal group, NS: no significance, by two-tailed Student's *t* test, *n* = 8, bar = 1 mm.

**Figure 4:**
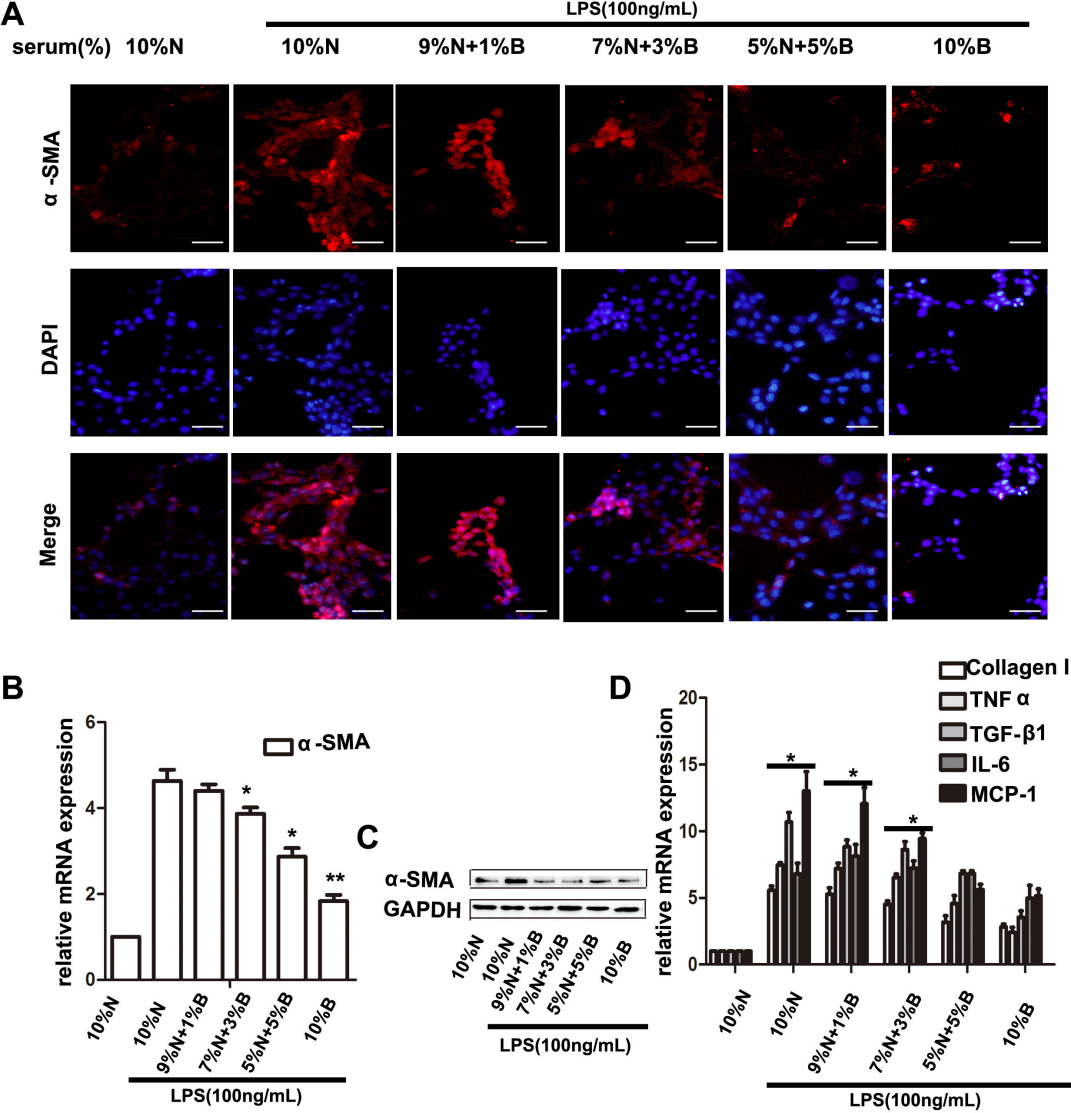
BBD-serum inhibited the LPS-induced HSCs activation Primary HSC was isolated and cultured with LPS and different gradient concentration of BBD-serum for 72 h. (**A**) Immunofluorescence staining was performed to identify the activated HSCs by α-SMA (× 200), bar = 100 um and the mRNA of α-SMA was determined by RT-PCR and western blot (**B** and **C**). (**D**) The collagen and inflammatory cytokines secreted by HSCs were also determined by RT-PCR. Compared to 10% N with LPS group, *P < 0.05; **P < 0.01, by two-tailed Student's *t* test, N: normal-serum; B: BBD-serum.

**Figure 5:**
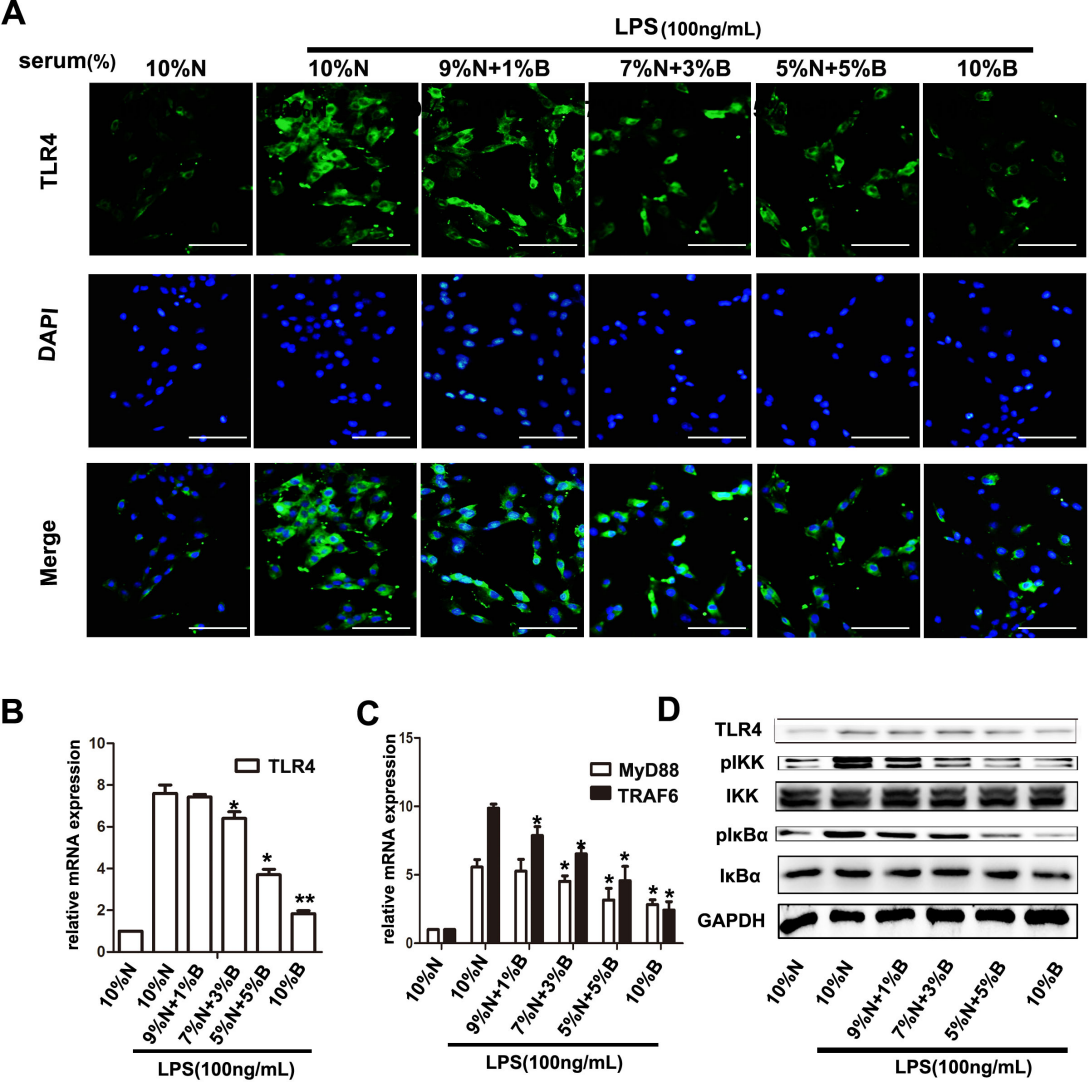
BBD-serum inhibited the LPS-induced HSCs activation by blocking NF-κB signaling pathway Primary HSC was isolated and cultured with LPS and different gradient concentration of BBD-serum for 72 h. (**A**) Immunofluorescence staining was performed to identify the expression of TLR4 (× 200), bar = 100 um and the mRNA of TLR4 was determined by RT-PCR (**B**). (**C**) Downstream genes of TLR4 were detected by RT-PCR. (**D**) Quantitative analysis of the Western blots for IKK and IκBα. Compared to 10% N. with LPS group, *P < 0.05; **P < 0.01, by two-tailed Student's *t* test; N: normal-serum; B: BBD-serum.

Hepatic stellate cells (HSCs), are demonstrated as predominant cell producing ECM and inflammatory cytokines, and promoting hepaic fibrosis in liver injury [7]. The characters of activation HSCs are the morphological transition to myofibroblast-like cells and expression of α- Smooth muscle actin (SMA) [14, 23]. We determined the effect of BBD on the expression of α-SMA by Immunohistochemical (IHC) assay. The result revealed that α-SMA expression was enormously elevated in DEN-group, and was suppressed following BBD treatment (Figure 3A). Meanwhile, the expression of α-SMA was also detected by RT-PCR and Western blot in corresponding liver fresh tissues (Figure 3B, 3C). DEN can disrupt the balance of intestinal flora and increase permeability of the intestinal mucosal barrier, which lead to a high concentration of LPS in portal vein [32]. As a result, high concentration of LPS aggravate hepatic fibrosis. Interestingly, compared to DEN-group, BBD did not significantly influence the level of LPS in portal vein analyzed by Elisa assay kit (Figure 3D). Furthermore, the expression of TLR4 was detected by IHC staining. As the figures indicated, BBD can obviously down-regulate the expression of TLR4 in rat livers insulted by DEN (Figure 3E). At the same time, the expression of TLR4 was also analyzed by RT-PCR and Western blot, accordingly (Figure 3F, 3G). To investigate the mechanism of BBD inhibiting HSCs activation, primary HSCs were isolated as previously described [17] and cultured in plastic cell culture dishes. In addition, we collected portal vein serum from normal rats (normal-serum) and BBD treated rats (BBD-serum). In the LPS-induced HSCs activation system *in vitro*, BBD-serum was added at the concentration of 1%, 3%, 5% and 10%, the rest of serum supplied by normal-serum to keep the serum concentration at 10% (v/v).72 h later, cellular immunoflurescence (IF) staining was performed. Compared with only LPS-activated HSCs group, BBD-serum could significantly inhibit HSCs activation by staining α-SMA and the inhibition effect was dose-dependent (Figure 4A). In addition, the mRNA and protein expression of α-SMA were detected (Figure 4B, 4C). And the mRNA expression of collagen I and inflammatory cytokines such as IL-6, TGF-β1, TNF-α and MCP-1 had the consistent consequence (Figure 4D). These results indicated that BBD-serum, which contained bioactive components, inhibited HSCs activation and pro-fibrotic cytokines expression. Furthermore, the expression of TLR4 on HSCs was also stained by IF assay (Figure 5A), The result was consistent with the above results. In addition, the RT-PCR results indicated that TLR4 inhibited by Myd88-depented pathway, and TRAF6, the downstream gene of TLR4, was also inhibited. (Figure 5B, 5C). To further demonstrate the mechanism of BBD-serum on HSCs activation, the IκBα/NF-κB signal pathway was analyzed. IκBα/NF-κB system is considered a major intracellular inflammatory pathway, mediating the inflammatory responses [24]. In HSCs, Nuclear Factor kappa B (NF-κB) plays an important role in regulation of cellular functions, including transition to myofibroblastic-like cell, and secretion of α- SMA and inflammatory cytokines. Inhibition of NF-κB pathway can significantly suppress inflammation and fibrosis [25, 26]. LPS increased NF-κB expression in HSCs significantly through upstream IKK pathway which was assessed by measurement of p-IκBα protein expression. Our study demonstrated that compared to HSCs induced by LPS only, BBD-serum markedly decreased the expression of p- IKK and p-IκBα in HSCs at 72 h (Figure 5D).

### BBD-serum could also suppress the proliferation of HSCs via ERK/Cyclin D1 pathway

**Figure 6:**
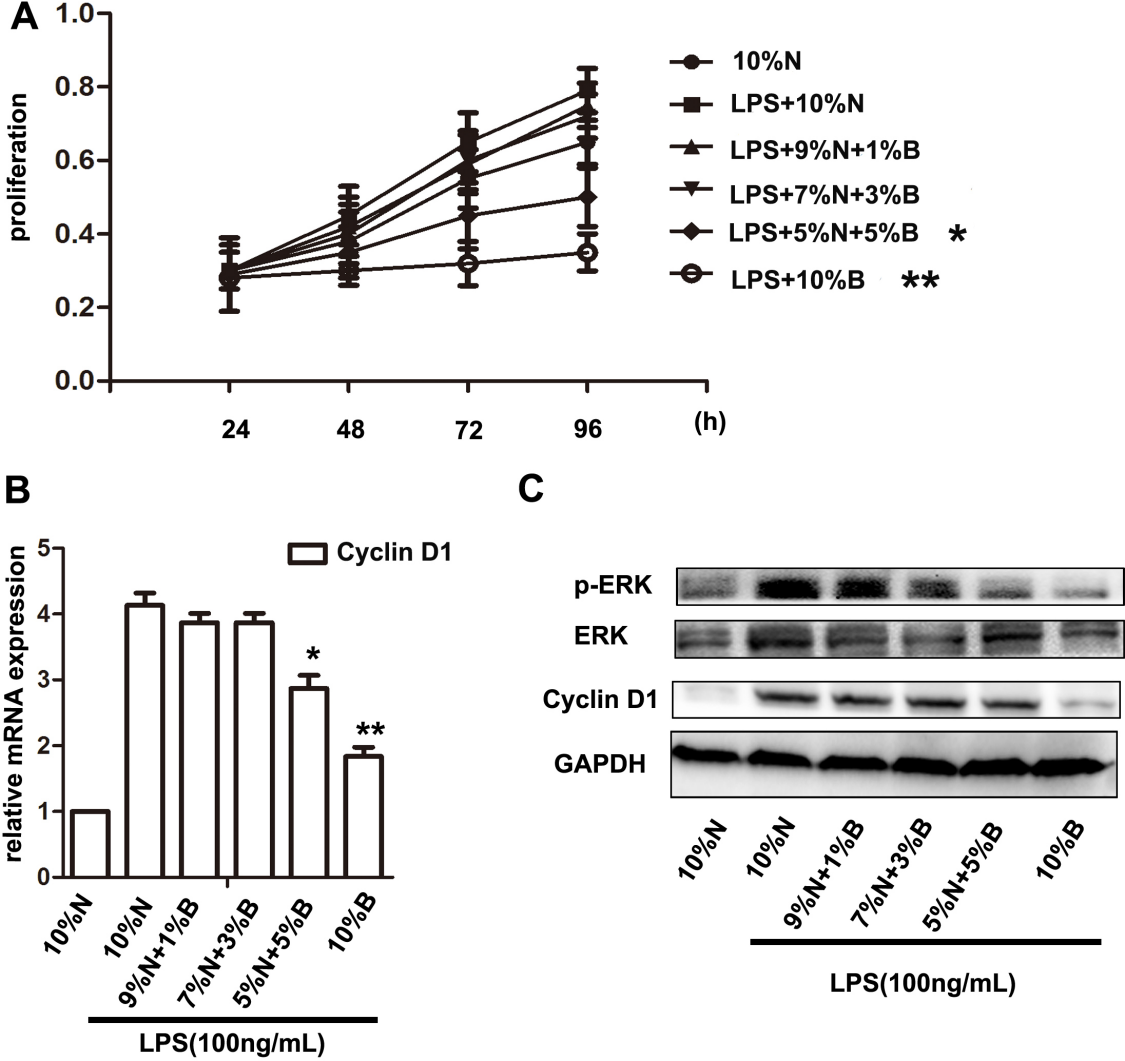
BBD-serum inhibited the LPS-induced HSCs proliferation by ERK signaling pathway (**A**) LPS-induced HSCs were treated with a gradient concentration of BBD-serum, and CCK-8 was performed to measure the proliferation at 24 h, 48 h, 72 h and 96 h. (**B**) The expression of Cyclin D1 in HSCs after administrated a different ratio BBD-serum by RT-PCR at 72 h. (**C**) Western blot was used to detect ERK and Cyclin D1 expressed in LPS-induced HSCs after treatment with BBD-serum at 72 h. Compared to10% N. with LPS group,*P < 0.05, **P < 0.01; by two-tailed Student's *t* test; N: normal-serum; B: BBD-serum.

At the same time, to determine the effects of BBD-serum on inhibition of HSCs proliferation, CCK-8 assay was performed. The optical density (OD) value was detected at 2 h after adding reagents and calculated the cell proliferation accordingly. The result of CCK-8 measurement indicated that the proliferation of HSCs could be facilitated by LPS and significantly suppressed with 5% and 10% BBD-serum (v/v) administration (Figure 6A). Present studies have demonstrated that the proliferation of LPS-induced HSCs can be facilitated by c-myc or Cyclin D1 induced by extracellular-related kinase (ERK) signaling pathway [17–20]. LPS can up-regulate the expression of phosphorylation of extracellular-related kinase (p-ERK) via TLR4 signaling pathway. Then we cultivate primary HSCs and treated LPS meanwhile BBD-serum was administrated with gradient concentration for 72 h. The results indicated that BBD-serum blocked LPS-induced HSCs proliferation through inhibiting ERK/Cyclin D1 signaling pathway determined at 72 h by RT-PCR and Western Blot (Figure 6B–6C).

## DISCUSSION

In this study, we showed the therapeutic function of Babao Dan (BBD) in DEN-induced rat hepatic fibrosis and explored the possible mechanisms. We demonstrated BBD can significantly reduce the hepatic injury and inflammatory infiltration, at the same time, inhibited the deposition of extracellular matrix (ECM) and the generation of myofibrocytes, which we considered them come from hepatic stellete cells (HSCs) activation. In addition, we demonstrated that BBD down-regulated the expression of TLR4 in rat livers, and had no effect on the concentration of lipopolysaccharide (LPS). *In vitro* study, BBD-serum can obviously inhibit LPS-induced HSCs activation and TLR4 expression. Furthermore, we illustrated the mechanism of BBD inhibited HSCs activation by NF-κB signaling pathway and blocked HSCs proliferation by ERK signaling pathway. Moreover, we found that BBD had no obvious side effect on normal rat livers. This result indicated that BBD may be a potential therapeutic choice to cure hepatic fibrosis.

Hepatic fibrosis is a chronic damage-repairing process, characterized by the deposition of ECM [1–3]. Advanced fibrosis led to cirrhosis, which is an irreversible progressing and often following severely complication, such as portal hypertension, liver function failure, high susceptibility to infection or to developed hepatocellular carcinoma (HCC) [4–6]. Hepatic stellate cells (HSCs) are considered as the predominant producers of fibrotic ECM during liver fibrosis [7, 8].

In cirrhotic rats or patients, gastrointestinal tract produces and absorbs considerable bacterial LPS with increased permeability of the intestinal mucosal barrier [13]. Meanwhile, liver's ability to detoxify bacterial endotoxins is also damaged. As a result, LPS, located in the cytoderm of Gram-negative bacteria, enormously induce HSCs activation and aggravate liver fibrosis, which has been proved to be an important mechanism in liver injury [14]. The mechanism of LPS increased hepatic fibrosis is by increasing the expression of TLR4. TLR4 is an innate immune receptor that can be enormously increased by LPS mediated by lymphocyte antigen 96 also known as MD-2 [11]. TLR4 signaling leads to activation of nuclear factor kappa B (NF-κB), an ubiquitous eukaryotic proinflammatory transcription factor which is noncovalently bound to cellular inhibitor of κB (IκBα) [27]. LPS can stimulate IκBα kinases activation that phosphorylate IκBα (p- IκBα), causing subsequent IκBα ubiquitination and proteasomal degradation. consequently, the NF-κB system is considered a major intracellular inflammatory pathway, mediating most of the inflammatory responses [24]. After IκBα released from the IκB/NFκB complex, the rest known as p65, then translocates into the nucleus, where it initiates the expression of inflammation genes and further participates in the tissue repair and remodeling processes [28, 29]. Meanwhile, LPS can up-regulated the expression of phosphorylation of extracellular-related kinase (p-ERK), which has demonstrated in facilitating HSCs proliferation by c-myc or Cyclin D1 [30, 31]. Furthermore, inhibit TLR4 or TLR4 mutation can obviously down-regulate inflammation and fibrosis [15, 21, 22]. However, there is still not any useful drugs to cure hepatic fibrosis and without obviously side effects.

**Figure 7:**
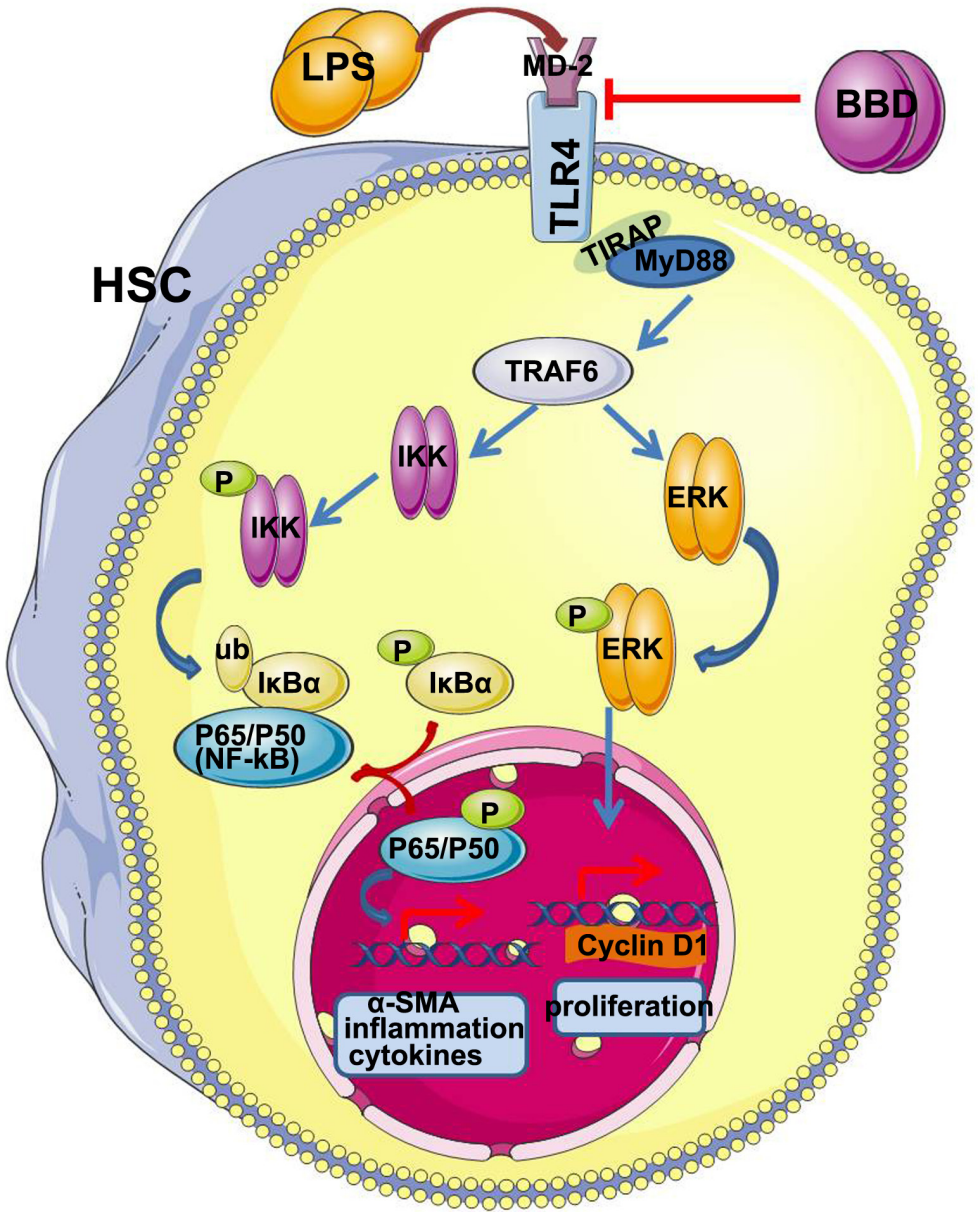
A proposed anti-fibrosis signaling pathway of BBD BBD inhibits upstream signal TLR4 expression, and via MyD88 dependent pathway in LPS-induced HSCs. In addition, BBD-serum can down-regulated the expression of α-SMA and inflammatory cytokines throughTLR4/NF-κB pathway. At the same time, BBD can inhibit HSCs proliferation via TLR4/ERK pathway. The element was obtained from Servier Medical Art (http://www.servier.com/servier-medical-art) and the figure was designed and drew by Lei Liang.

In recent decades, traditional Chinese medicine has been widely used as a complementary and alternative medicine to treat chronic liver diseases. BBD, a Chinese traditional medicine, has been widely used in clinic. So we want to explore the effect of BBD on fibrosis and research the possible mechanisms. In our study, we demonstrated that BBD had a therapeutic role in DEN-induced hepatic injury by testing ALT and AST. Meanwhile, Histological examination, mRNA expression and ELISA detection indicated that BBD can inhibit hepatocyte injury and inflammatory cytokines expression. Then, we analyzed hepatic fibrosis by Masson staining, hydroxyproline detection and Siris red staining. As the result demonstrated that BBD could obviously attenuate hepatic fibrosis induced by DEN. In addition, the expression of α-SMA, a marker of activated HSCS, indicated that BBD could inhibit HSCs activation. Present study has demonstrated DEN can disrupt the balance of intestinal flora and increase permeability of the intestinal mucosal barrier, which lead to a high concentration of LPS in portal vein, and LPS could aggravate hepatic fibrosis [32]. So we analyzed the concentration of lipopolysaccharide (LPS) in portal vein. Interestingly, BBD did not affect the concentration of LPS in portal vein. Subsequently, immunohistological staining, RT-PCR and Western blot all presented that BBD can down-regulated the expression of TLR4 and α-SMA. The result showed that BBD played role in attenuating fibrosis not by inhibited the absorption of LPS, but through blocking TLR4 signaling pathway directly. Moreover, the process of hepatic stellate cells activated by LPS can be suppressed by BBD-serum *in vitro* by IF staining and analyzed the mRNA expression of α-SMA, collagen I and other inflammatory cytokines, such as IL-6, TGF-β1,TNF-α and MCP-1. In addition, we identified BBD-serum can block the expression of TLR4 induced by LPS through Myd88 dependent pathway. Furthermore, we demonstrated that BBD inhibited the HSCs activation and proliferation by suppressed TLR4 signaling pathway, and further research indicated that they may be through TLR4/NF-κB and TLR4/ERK pathway, respectively. In conclusion, BBD can be used as a potential therapeutic choice to cure hepatic fibrosis. However, the monomer of BBD bioactive components need be extracted and further research (Figure 7).

## MATERIALS AND METHODS

### Reagents

BBD was obtained from Shanghai Pharmaceuticals Holding Co., Ltd, Shanghai, China, and dissolved in normal saline (NS). Diethylnitrosamine (DEN) was diluted in normal water at a concentration of 1:10,000 (v/v). Hydroxyproline Testing Kit (A030-2) was bought form Jiancheng, Nanjing, China. The antibody of α-smooth muscle actin (α-SMA) (ab5694, Abcam), Toll-like receptor 4 (TLR4) (ab22048, Abcam), pIKKα/β(2697, Cell Signaling), IKKα/β(sc-7607, Santa Cruz), pIκBα(2859, Cell Signaling), IκBα(4812, Cell Signaling), pERK1/2 (4370, Cell Signaling), ERK1/2 (4695, Cell Signaling), Cyclin D1 (ab134175,abcam) and GAPDH (AP0063, Bioworld) were used for western blot or cellular immunofluorescence staining. LPS (H178, Jiancheng, Nanjing, China), IL-6 (H007, Jiancheng, Nanjing, China), TGF-β1(H034, Jiancheng, Nanjing, China), TNF-α (H052, Jiancheng, Nanjing, China), MCP-1 (H115, Jiancheng, Nanjing, China) were used for endotoxin enzyme-linked immunosorbent assay (ELISA) test. LPS was purchased from Sigma.

### Animals

All Sprague-Dawley (SD) rats (male, 190 ± 15 g body weight) were purchased from the Shanghai Experimental Animal Center at the Chinese Academy of Sciences in Shanghai, China. The rats were housed under pathogen-free conditions and freedom to food and water and were randomly divided into four groups. Experiments and procedures were approved by the Animal Ethics Committee of the Second Military Medical University, Shanghai, China, and all experiments were performed in accordance with the approved guidelines and regulations.

### Methods

### Animal hepatic fibrosis model

Liver fibrosis model was induced in rat by drinking diethylnitrosamine (DEN) water at a concentration of 1:10,000 (v/v). BBD dissolved in normal saline and was gavaged in rats at a dosage of 0.5g/kg body weitht once every two days. For this model, 32 rats were randomly divided into four groups and treated as follows: the normal group (*n* = 8, normal water and gavaged NS), the BBD group (*n* = 8, normal water and gavaged BBD), the DEN group (*n* = 8, DEN-water and gavaged NS) and the DEN/BBD group (*n* = 8, DEN-water and gavaged BBD). All animals were sacrificed after 8 weeks.

### Serum biochemical analysis

Serum of rats was collected by centrifugation at 3000g, 4°C for 10 min. Alanine aminotransferase (ALT) and aspartate aminotransferase (AST) were determined in all experimental rats by a biochemical analyzer.

### Histological examination and immunohistological staining

4-μm-thick paraffin-embedded liver sections were stained with hematoxylin and eosin (H&E) for histopathological examination, the sections were stained with Masson'trichrome and Sirius red for collagen determintion. Connective tissues stained with Sirius red were measured on an image analyzer (Image-Pro Plus, Media Cybernetics) by a technician blinded to the samples for the semiquantitative analysis. To detect liver fibrosis quantitatively, 100 mg of wet liver samples were subjected to acid hydrolysis to determine the amount of hydroxyproline according to the protocol in the Hydroxyproline Testing Kit. Immunohistochemical examinations were carried out to determine the expression of α-SMA and TLR4. Paraffin sections used for immunohistochemistry assay were 4-μm thick, and the detail process is done according to method described before [30].

### Enzyme-linked immunosorbent assay

Concentration of LPS was detected by Rat ELISA. Samples were added into the test wells followed by ET-antibody and Streptavidin-HRP. Then seal the wells with the sealing membrance, and gently shaking, incubated 60 minutes at 37°C. After that, the wells was removed the memberance carefully, and drained the liquid, disgarded the remaining water. Then add chromogen solution A and B to each well and incubate for 10 min at 37°C away from light. Subsequently, stop solution was added into each well and measure the optical density (OD) under 450 nm wavelength in 10min then calculate the concentration according to the standard. ELISA was also performed to detected the secretion of IL-6, TGF-β1, TNF-α and MCP-1 according to the manufacturer instructions.

### Cell culture and treatment

Primary HSCs were freshly isolated from SD rats as previously described [17]. The primary HSCs were cultured with Roswell Park Memorial Institute (RPMI)-1640 supplemented with 2 mM L-glutamine (Sigma, St. Louis, MO), 10% fetal bovine serum (FBS), 100 ug/ml penicillin and 100 ug/ml streptomycin in the condition of 37°C with 5% CO_2_. Then we treated HSCs with 100 ng/ mL LPS (Sigma) supplemented with 10% serum(v/v) for 72 h, as 1% BBD-serum with 9% normal rat serum group, 3% BBD-serum with 7% normal rat serum group, 5% BBD-serum with 5% normal rat serum group and 10% BBD-serum group respectively.

### Real-time quantitative PCR

To test mRNA expression, total RNA from every frozen rat livers tissue and different group cells was extracted by using TRIZOL (Invitrogen, Carls-bad, CA, USA). Prime Script RT reagent Kit (Takara, Kyoto, Japan) was performed for cDNA synthesis. Relative quantitative PCR 9RT-PCR) was performed using SYBR Green PCR Kit (Applied BI) according to the manufacturer instructions. The quantitative analysis of relative mRNA was conducted with GAPDH as the reference. Primer sequences are listed in Table 1 as follows:

**Table 1:** The primer sequences of relative genes

Gene	Forward	Reverse
α *–SMA*	5′-ACTGGGACGACATGGAAAAG-3′	5′-CATCTCCAGAGTCCAGCACA-3′
*Collagen* I	5′-AGGCATAAAGGGTCATCGTG-3′	5′-ACCGTTGAGTCCATCTTTGC-3′
*IL-6*	5′-CCGGAGAGGAGACTTCACAG-3′	5′-ACAGTGCATCATCGCTGTTC-3′
*TGF-β1*	5′-ATACGCCTGAGTGGCTGTCT-3′	5′-TGGGACTGATCCCATTGATT-3′
*TNF-*α	5′-AGATGTGGAACTGGCAGAGG-3′	5′-CCCATTTGGGAACTTCTCCT-3′
*MCP-1*	5′-ATGCAGTTAATGCCCCACTC-3′	5′-TTCCTTATTGGGGTCAGCAC-3′
*TLR4*	5′-TGCTCAGACATGGCAGTTTC-3′	5′-TCAAGGCTTTTCCATCCAAC-3′
*Myd88*	5′-GAGATCCGCGAGTTTGAGAC-3′	5′-CTGTTTCTGCTGGTTGCGTA-3′
*TRAF6*	5′-AGGGTACAATACGCCTCACG-3′	5′-GCGGGTAGAGACTTCACAGC-3′
*Cyclin D1*	5′-GCGTACCCTGACACCAATCT-3′	5′-GGCTCCAGAGACAAGAAACG-3′
β *–actin*	5′-GCCAACACAGTGCTGTCTGG-3′	5′-TGATCCACATCTGCTGGAAGG-3′

### Cellular immunofluorescence staining

Different group cells were washed with PBS followed by 4% paraformaldehyde solution, then treated with 0.5% Tron-X 100 solution in PBS (v/v). After that, wash the cells with PBS and added antibodies α-SMA and TLR4 overnight at 4°C. Alexa Fluor 488 goat anti-mouse and anti-rabbit IgG secondary antibodies were used at a concentration of 1/200 correspondingly. Subsequently, nucleus was counter stained with DAPI at 1 μg/mL.

### Western blotting

The fresh liver tissue or treated cells were washed with PBS and lysed by RIPA and PMSF at a ratio of 100:1 to obtain the total protein for Western blot. Equal amount of proteins was separated by SDS-PAGE and transferred to polyvinylidene fluoride membrane. After transferring, the polyvinylidene fluoride membrane was blocked in 5% fat-free milk/1 × TBS/0.1% Tween-20 for 1 h at room temperature and then incubated with primary antibodies with gentle agitation overnight at 4°C. And then, the membrane was washed with 1 × TBS/0.1% Tween-20 before incubated with the secondary antibody according to the primary antibody for 1 h at room temperature. Immunoblots were performed by using the BeyoECL Plus substrate system (Beyotime), followed by washing with 1 × TBS/0.1% Tween-20. Western blot analyzed of α-SMA, TLR4, pIKKα/β, IKKα/β, pIκBα, IκBα, pERK1/2, ERK1/2, Cyclin D1 and GAPDH.

### Measurement of HSCs proliferation

3 × 10^3^ activated HSCs were plated in triplicate wells on a 96-well plate and cultured for 24 h. These cells were then treated with 100 ng/mL LPS (Sigma) supplemented with RPMI-1640 contained different ratio BBD-serum and normal rat serum as the ratio mentioned before. Subsequently, the OD value was detected by Cell Counting Kit-8 (DOJINDO, Tokyo, Japan) assays. The reagent was added at 0 h, 24 h, 48 h, 72 h and 96 h. Then, the OD value was detected at 2 h after the reagent added.

### Statistical analysis

Results are presented as the mean of three independent experiments (mean ± SD). The two-sided independent Student's *t* test was performed to analyze the differences in hepatic function indicator, hydroxyproline content, and gene expression levels. *P* < 0.05 was considered statistically significant.
